# Pathology of Cutaneous T Cell Lymphoma: A Narrative Review

**DOI:** 10.3390/cancers18071169

**Published:** 2026-04-04

**Authors:** Ting Zhang, Yi Hu, Kexin Wang, Zhaohuai Zhang, Ying Wang, Yu Zhang, Zuotao Zhao

**Affiliations:** 1 Department of Dermatology, Tianjin Institute of Integrative Dermatology, Tianjin Academy of Traditional Chinese Medicine Affiliated Hospital, Tianjin 300193, China; tingzhang1113@163.com; 2 Graduate School, Tianjin University of Traditional Chinese Medicine, Tianjin 301617, China; huyi_doc@outlook.com (Y.H.); bunao331@163.com (K.W.); 3 Department of Immunology, School of Basic Medical Sciences, Peking University, Beijing 100191, China; zh_zhang@pku.edu.cn (Z.Z.); yw@bjmu.edu.cn (Y.W.); 4 NHC Key Laboratory of Medical Immunology, Peking University, Beijing 100191, China; 5 Medicine Innovation Center for Fundamental Research on Major Immunology-Related, Diseases, Peking University, Beijing 100871, China; 6 Center for Human Disease Genomics, Peking University, Beijing 100871, China

**Keywords:** cutaneous T-cell lymphoma, pathophysiological mechanisms, JAK/STAT signaling pathway, tumor microenvironment, epigenetic landscape

## Abstract

Cutaneous T-cell lymphoma (CTCL) comprises a heterogeneous group of extranodal non-Hodgkin lymphomas. With the publication of the fifth edition of the World Health Organization Classification of Hematolymphoid Tumors, the diagnostic framework for CTCL has shifted from primarily morphologic phenotypes toward an emphasis on molecular drivers. Current research suggests that malignant clones may arise from somatic mutations at the hematopoietic stem cell stage and may follow a continuous hematogenous dissemination model with bidirectional trafficking between the skin and systemic circulation. At the molecular level, genomic instability, often associated with somatic copy-number variations, may promote activation of the janus kinase-signal transducer and activator of transcription (JAK/STAT) signaling pathway through gene-dosage effects. In parallel, chromatin remodeling linked to EZH2 overexpression and reduced special SATB1 expression may support a Th2-polarized program. This phenotype may contribute to epidermal barrier impairment via cytokines such as Interleukins-4 (IL-4) and IL-13, potentially creating conditions permissive for *Staphylococcus aureus* colonization. Microbial superantigens and exotoxins may further contribute to tumor progression and therapeutic resistance by reinforcing JAK/STAT signaling, particularly STAT3, and reducing CD8+ T-cell–mediated immune surveillance. In the dermis, reprogramming of cancer-associated fibroblasts and polarization of macrophages toward an M2 phenotype may collectively contribute to an immunosuppressive niche. Emerging biomarkers, including CD74, and acquired resistance mechanisms after anti-C-C chemokine receptor 4 therapy further extend the translational relevance of recent pathologic findings. Overall, CTCL evolution appears to be a systemic process shaped by interactions between tumor-intrinsic genetic alterations and the skin microenvironment.

## Simple Summary

Cutaneous T-cell lymphoma (CTCL) is a rare cancer of T cells that often starts in the skin but can involve the blood and lymph nodes. Early symptoms may resemble common skin diseases, which can delay diagnosis and treatment. This review summarizes evidence that CTCL may begin from early blood-forming cells in the bone marrow, then spread between skin and blood over time. We explain how genetic and epigenetic changes activate growth signals, and how the skin environment—barrier damage, *Staphylococcus aureus* colonization, and immune-suppressing cells—can support disease persistence. Emerging biomarkers such as CD74 and acquired resistance after anti-C-C chemokine receptor 4 therapy further highlight the relevance of pathologic mechanisms to diagnostic and therapeutic applications. Overall, CTCL appears to be a systemic disease shaped by both tumor-internal changes and the skin microenvironment.

## 1. Introduction

Cutaneous T-cell lymphoma (CTCL) comprises a heterogeneous group of extranodal non-Hodgkin lymphomas characterized by preferential skin homing and clonal expansion of malignant T lymphocytes within the cutaneous microenvironment [1,2]. CTCL accounts for approximately 75–80% of all primary cutaneous lymphomas [3]. The disease spans an indolent-to-aggressive spectrum, ranging from mycosis fungoides (MF) to more aggressive entities, such as Sézary syndrome (SS). The WHO-HAEM5, published in 2022, shifted the diagnostic approach to CTCL from conventional morphologic assessment toward an integrated model incorporating molecular features [4].

Although genomic studies have advanced the molecular characterization of CTCL, important gaps remain. Existing reviews have focused primarily on advanced-stage disease, whereas the molecular distinction between early-stage CTCL and chronic inflammatory dermatoses remains poorly defined. Limited comparative data may contribute to delayed diagnosis in clinical practice [5]. In addition, the conventional mature memory T-cell model of CTCL ontogeny is being reconsidered in light of alternative hypotheses, including a hematopoietic stem cell (HSC) origin and the circulating malignant clone model [6]. These concepts have not yet been systematically incorporated into current diagnostic frameworks. The mechanisms underlying acquired resistance to targeted agents, including mogamulizumab, also require further pathologic investigation.

Recent advances in single-cell transcriptomics and high-throughput sequencing have refined current understanding of CTCL pathogenesis, with increasing attention to epigenetic regulation and interactions within the TME. Accordingly, a systematic assessment of progress over the past five years is warranted. This review summarizes current progress in the molecular pathogenesis of CTCL, with particular emphasis on disease ontogeny, dysregulated signaling pathways, microenvironmental interactions, and mechanisms of therapeutic resistance. It also addresses challenges in diagnosis, including the masking effect associated with biologic agents such as dupilumab, and assesses the translational relevance of emerging targets such as CD74. Collectively, these issues are relevant to ongoing efforts to refine diagnostic classification and therapeutic stratification in CTCL.

## 2. Classification and Pathophysiologic Evolution of Cutaneous T-Cell Lymphoma

The WHO-HAEM5 classification (2022) reflects a shift in CTCL diagnosis from morphologic assessment alone to an integrated approach that incorporates molecular alterations (Table 1) [7]. Mycosis fungoides (MF), the most common CTCL subtype, generally shows a pathophysiologic course that parallels the clinical progression from patch to plaque and ultimately to tumor stage. At the molecular level, this progression is often accompanied by loss of pan-T-cell markers (e.g., CD7 and CD26) and persistent JAK/STAT pathway activation [8,9,10]. Sézary syndrome (SS) is characterized by generalized erythroderma and peripheral blood involvement. The malignant clone is capable of trafficking among the skin, blood, and lymphatic compartments and commonly exhibits chromosomal instability and mutations in genes such as MYC proto-oncogene, bHLH transcription factor and tumor protein p53 (*TP53*) [11,12]. In less common subtypes, such as subcutaneous panniculitis-like T-cell lymphoma (SPTCL), diagnosis is increasingly informed by subtype-associated genetic findings, such as germline HAVCR2 mutations, which are associated with immune dysregulation and hemophagocytic lymphohistiocytosis [13,14].

In early CTCL, malignant clones may closely resemble benign inflammatory dermatoses such as atopic dermatitis (AD) in both clinical presentation and Th2-skewed immune profiles, which may contribute to delays in diagnosis [15]. Both conditions may show predominant CD4+ T-cell infiltration and increased expression of cytokines such as IL-4 and IL-13, making early CTCL difficult to distinguish from AD on clinical and immunologic grounds [16]. Clinical observations indicate that some patients initially diagnosed with AD subsequently develop rapid disease progression or progression from a previously unrecognized subclinical state to clinically apparent disease after initiation of dupilumab therapy [17,18]. Proposed mechanisms include compensatory changes in IL-13 signaling, disruption of local Th1/Th2 balance, and reduced tumor immune surveillance, raising the possibility that such treatment may unmask or permit expansion of previously unrecognized malignant clones [17,19,20].

Differences in phenotype and treatment response across CTCL subtypes are closely associated with the molecular profiles of malignant cells. For example, aberrant CD74 expression has been implicated in immune evasion and tumor progression through activation of survival-related signaling pathways [21]. In addition, distinct expression patterns of chemokine receptors, such as C-C chemokine receptor 4 (CCR4), CCR7, and CD62L, may influence whether malignant clones remain confined to the skin or acquire the capacity for systemic dissemination [22,23]. Classification based on cellular and molecular features also provides a framework for examining the cellular origin of CTCL—whether malignant transformation arises from mature peripheral memory T cells or from genetic instability arising at the HSC stage.

**Table 1:** Classification and Characteristics of Cutaneous T-Cell Lymphoma Subtypes.

Category	Representative Entities	Clinical Features and Prognosis	Key Immune Markers/ Molecular Features	References
Classic CTCL	Mycosis fungoides	Chronic course; evolves through patch, plaque, and tumor stages; diagnosis relies on clinicopathologic correlation.	Mature helper T-cell phenotype (CD3+, CD4+, CD8−); common loss of CD7 and downregulation of CD2/CD3/CD5; large cell transformation in tumor stage.	[4,9,10]
Leukemic Subtypes	Sézary syndrome	Aggressive leukemic subtype; triad of erythroderma, lymphadenopathy, and peripheral blood Sézary cells; poor prognosis.	CD4/CD8 ratio > 10; CD4+ CD7− ≥ 40% or CD4+ CD26− ≥ 30%; high expression of CCR7 and CD62L; MYC amplification and CDKN2A/B loss.	[11,12,24,25]
CD30+ LPDs	Lymphomatoid papulosis	Self-limiting recurrent papules; clinical spectrum from benign to low-grade malignancy; clinicopathologic discordance.	High CD30 expression; subset with DUSP22 or TP63 rearrangements.	[26,27,28]
CD30+ LPDs	Primary cutaneous anaplastic large cell lymphoma	Solitary nodules or ulcers; favorable prognosis; histological sheets of large cells.	CD30+ (>75%); 20–30% with DUSP22/IRF4 rearrangements (biphasic pattern); anaplastic lymphoma kinase protein typically absent.	[29,30]
Panniculitis-like	Subcutaneous panniculitis-like T-cell lymphoma	Localized to subcutaneous fat; subcutaneous nodules; associated with hemophagocytic lymphohistiocytosis.	CD8+; αβ T-cell phenotype; germline HAVCR2 (TIM-3) mutations.	[13,14]
Rare Aggressive Subtypes	CD8+ aggressive epidermotropic cytotoxic T-cell lymphoma	Ulceration and necrosis; rapid progression; extremely poor prognosis.	CD8+; Granzyme B+; common JAK2 fusions or STAT3/STAT5B activating mutations.	[31,32]
Rare Aggressive Subtypes	Primary cutaneous γδ T-cell lymphoma	Involvement of epidermis, dermis, and subcutis; extremely poor prognosis.	γδ TCR+; CD4−/CD8−; high expression of cytotoxic molecules; frequent aberrant activation of the JAK pathway.	[4,31]

## 3. Cellular Origin and Evolution of Cutaneous Malignant T Cells

**Figure 1:**
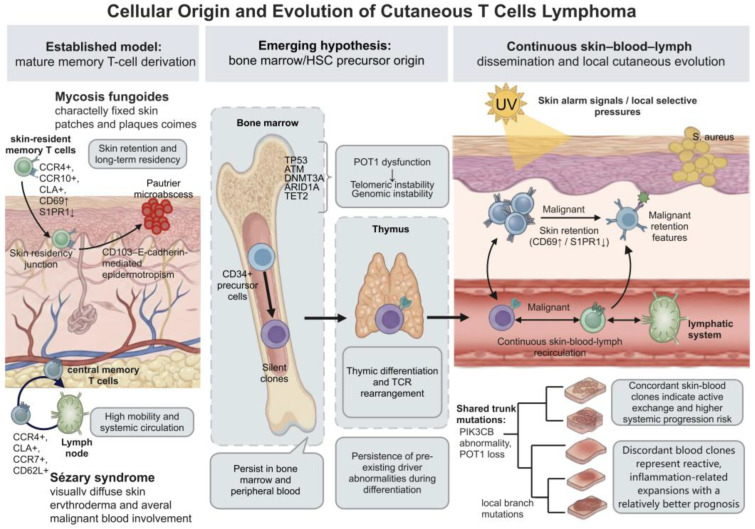
Cellular origins and clonal progression of cutaneous T-cell lymphoma. In the mature cell-of-origin model, mycosis fungoides is proposed to arise from skin-resident T cells, whereas Sézary syndrome is linked to central memory T cells. An emerging model suggests a hematopoietic stem cell precursor origin, with early driver abnormalities acquired in the bone marrow retained after thymic differentiation. Malignant clones circulate among the skin, blood, and lymphatic system and may undergo branched evolution under local selective pressures, including ultraviolet exposure and *Staphylococcus aureus*. Concordant skin-blood clones are associated with a higher risk of progression, whereas discordant blood clones are associated with a better prognosis. ARID1A: AT-rich interaction domain 1A; *ATM*: Ataxia-telangiectasia mutated; CCR: C-C chemokine receptor 4; CD: Cluster of differentiation; CLA: Cutaneous lymphocyte-associated antigen; DNMT3A: DNA methyltransferase 3 alpha; HSC: Hematopoietic stem cell; PIK3CB: Phosphatidylinositol-4,5-bisphosphate 3-kinase catalytic subunit beta; *POT1*: Protection of telomeres 1; *S. aureus*: *Staphylococcus aureus*; S1PR1: Sphingosine-1-phosphate receptor 1; TCR: T-cell receptor; TET2: Tet methylcytosine dioxygenase 2; *TP53*: Tumor protein p53; UV: Ultraviolet.

The cellular origin of CTCL is central to current models of disease heterogeneity and clinical behavior (Figure 1). Classical models attribute the differences between MF and SS to distinct homing programs of skin-resident memory T cells (TRM) and central memory T cells (TCM). However, recent genomic evidence suggests that some malignant clones may originate before the mature peripheral T-cell stage, arising from early mutational events in bone marrow-derived hematopoietic stem cells (HSCs). This shift in emphasis from transformation of mature T-cell subsets to evolution at the progenitor stage may help explain the systemic dissemination and multisite involvement observed in CTCL.

### 3.1. Established Models: Mature Memory T-Cell Models and Chemokine Receptor Profiles

In the mature memory T-cell model, MF is generally considered to arise from malignant transformation of TRM cells and to retain persistent skin tropism and tissue residence as central biologic features [33]. Under physiologic conditions, these cells are recruited from the circulation to the skin through the coordinated action of chemokine receptors CCR4 and CCR10 and cutaneous lymphocyte-associated antigen (CLA). TRM cells achieve tissue residency through sustained CD69 expression, which promotes internalization and degradation of sphingosine-1-phosphate receptor 1 (S1PR1), thereby limiting responsiveness to the circulating S1P gradient [34,35]. Malignant MF clones appear to retain key features of this trafficking program, including migration toward the papillary dermis mediated by CCR4 and its ligands CCL17 (C-C motif chemokine ligand 17) and CCL22. Their characteristic epidermotropism is mediated in part by binding of the integrin CD103 (αEβ7) to E-cadherin on keratinocytes, a process associated histologically with Pautrier microabscess formation [3,36]. In the absence of S1PR1 expression, malignant cells show reduced egress from the skin and tend to present as relatively fixed patch or plaque lesions [37].

In contrast, SS is more consistent with a TCM-like cell of origin. SS cells not only express skin-homing receptors such as CCR4 and CLA but also retain lymph node-homing receptors, including CCR7 and L-selectin (CD62L) [38,39]. This combination of homing receptors enables SS cells to circulate among the skin, blood, and lymphatic compartments and may underlie systemic dissemination. This recirculating phenotype corresponds to the clinical triad of SS: diffuse erythroderma, lymphadenopathy, and leukemic involvement of the peripheral blood [40].

These differences in cellular origin are also reflected in the natural history and prognosis of MF and SS. MF typically follows a chronic course, with 5-year survival exceeding 95% in early-stage disease (IA–IIA). However, prognosis worsens with large cell transformation (LCT) or progression to tumor-stage disease (IIB or higher) [41,42]. In contrast, SS generally follows a more aggressive clinical course and often presents at stage III or IV. Reported 5-year survival rates range from approximately 24% to 36%, with median overall survival of 2.4 to 5 years [43]. Together, these observations support a pathobiologic basis for the differences in clinical progression and survival between MF and SS.

### 3.2. Emerging Hypotheses: Bone Marrow Origin and Hematogenous Seeding

The bone marrow origin hypothesis proposes that early oncogenic events in CTCL may arise in HSCs or multipotent lymphoid progenitors within the bone marrow. Supporting evidence comes from genomic analyses of CD34+ hematopoietic progenitors in patients with SS. These studies have identified somatic mutations in immature progenitors that are also present in mature peripheral tumor cells. The affected genes include *TP53*, *ATM*, DNA methyltransferase 3 alpha, AT-rich interaction domain 1A, and TET2, many of which are involved in tumor suppression or epigenetic regulation [6]. This finding suggests that progenitor cells may harbor early oncogenic alterations before thymic differentiation and antigen-specific T-cell receptor (TCR) rearrangement. Subsequently, these progenitors carrying early driver aberrations may enter the thymus, undergo TCR rearrangement during differentiation, and eventually give rise to dominant malignant clones within the skin microenvironment [44].

Among these early events, aberrations in protection of telomeres 1 (*POT1*) are of particular pathobiologic interest. *POT1* is a core member of the shelterin complex that recognizes and binds single-stranded telomeric DNA, thereby preventing chromosome ends from being recognized as sites of DNA damage [45]. Loss or dysfunction of *POT1* at the CTCL progenitor stage may promote telomeric instability, large-scale chromosomal rearrangements, and persistent genomic instability through the breakage-fusion-bridge cycle [46,47,48]. This may generate a clinically occult clonal population carrying early oncogenic events in the absence of recognizable tissue infiltration. Such progenitor cells may persist in the bone marrow and peripheral circulation for prolonged periods [49] and may show enhanced responsiveness to survival signals, including IL-7 and IL-15, and to chemokines such as CCL17 and CCL22 [50,51]. In the setting of skin barrier injury induced by ultraviolet (UV) exposure, chronic inflammation, or *Staphylococcus aureus* colonization, these cells may undergo activation and seed multiple skin sites through ongoing hematogenous dissemination, thereby promoting clonal expansion and progression toward overt malignancy [49].

Based on these findings, a model of continuous hematogenous seeding supports CTCL, particularly MF, as a disease involving ongoing exchange among the skin, blood, and lymphatic compartments [52]. This model suggests that malignant cell trafficking is not confined to late-stage disease but may occur throughout the disease course. Using high-sensitivity TCR sequencing, including the immunoSEQ platform, investigators have identified blood clones with distinct prognostic implications [53]. A dominant blood clone convergent with the cutaneous clone suggests active skin-blood exchange and is associated with a higher risk of systemic progression, whereas a dominant blood clone divergent from the cutaneous clone likely reflects reactive expansion in response to inflammatory stimuli and is associated with a more favorable prognosis. Branched evolutionary analyses further suggest that malignant clones in different lesions share progenitor-stage trunk mutations, such as PIK3CB aberrations or *POT1* loss, but acquire distinct branch mutations under local selective pressures, including UV exposure or chronic infection [54,55,56,57,58]. CTCL therefore shows relative consistency in early clonal markers across anatomic sites, followed by substantial genetic heterogeneity during subsequent subclonal expansion.

## 4. Genomic Alterations and Epigenetic Remodeling

**Figure 2:**
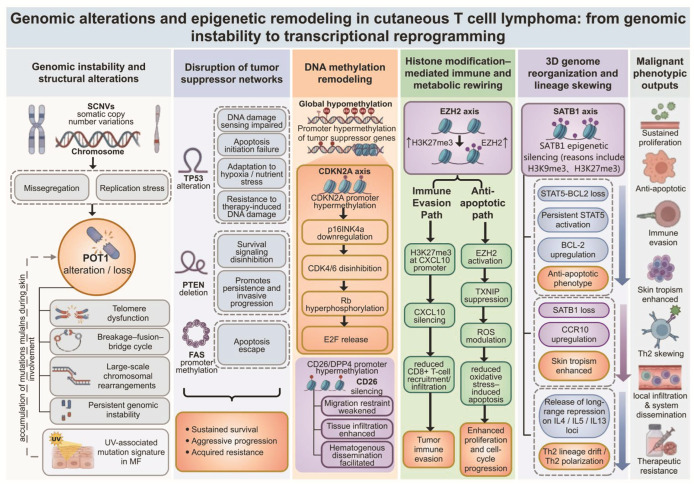
Genomic and epigenetic landscapes of cutaneous T-cell lymphoma. Genomic instability in cutaneous T-cell lymphoma involves somatic copy number variations, replication stress, chromosomal abnormalities, and *POT1* alteration or loss, which are linked to telomere dysfunction, chromosomal rearrangements, and persistent genomic instability. Disruption of tumor suppressor networks, including *TP53* alteration, PTEN deletion, and FAS promoter methylation, is associated with impaired DNA damage responses, apoptosis escape, and invasive progression. DNA methylation remodeling includes global hypomethylation and promoter hypermethylation of tumor suppressor genes, including the *CDKN2A* and CD26/DPP4 axes. Histone modification-mediated rewiring centered on EZH2 may contribute to immune evasion and apoptosis resistance. In parallel, 3D genome reorganization involving SATB1 is linked to persistent STAT5 activation, *BCL-2* upregulation, CCR10 expression, and Th2-skewed transcription. Together, these alterations are associated with proliferation, apoptosis resistance, dissemination, and therapeutic resistance. *BCL-2*: B-cell lymphoma 2; CCR10: C-C chemokine receptor 10; CD: Cluster of differentiation; *CDKN2A*: Cyclin-dependent kinase inhibitor 2A; CXCL10: C-X-C motif chemokine ligand 10; DNA: Deoxyribonucleic acid; DPP4: Dipeptidyl peptidase 4; E2F: E2F transcription factor; EZH2: Enhancer of zeste homolog 2; FAS: Fas cell surface death receptor; H3K9me3: Trimethylation of lysine 9 on histone H3; H3K27me3: Trimethylation of lysine 27 on histone H3; IL: Interleukin; MF: Mycosis fungoides; *POT1*: Protection of telomeres 1; PTEN: Phosphatase and tensin homolog; Rb: Retinoblastoma protein; ROS: Reactive oxygen species; SATB1: Special AT-rich sequence-binding protein 1; SCNVs: Somatic copy number variations; STAT5: Signal transducer and activator of transcription 5; Th2: T helper 2; *TP53*: Tumor protein p53. TXNIP: Thioredoxin-interacting protein.

The clonal evolution and progression of CTCL reflect the interplay between genomic instability and epigenetic remodeling (Figure 2). The genomic landscape is characterized primarily by somatic copy number variations (SCNVs), together with broad changes in chromatin organization. Genomic abnormalities contribute to dysregulation of the cell cycle and tumor suppressor networks, whereas aberrant DNA methylation, altered histone modifications, and reorganization of three-dimensional genomic architecture further reshape the transcriptional state of malignant T cells and their interactions with the microenvironment.

### 4.1. Genomic Structural Variations and Mutational Signatures

Studies suggest that pathogenic SCNVs account for approximately 92% of putative driver alterations in CTCL [59]. These widespread structural abnormalities are typically associated with chromosome segregation errors or replication stress. Telomere dysfunction resulting from *POT1* aberrations is thought to represent an early molecular event that may promote large-scale chromosomal rearrangements [60]. Mutational signature analysis further highlights the contribution of environmental exposure to clonal evolution. In MF, characterized by ultraviolet (UV)-induced C > T transitions at dipyrimidine sites, predominates and contributes approximately 52% of the mutational burden [61]. Although SS is dominated by age-related spontaneous deamination signatures, UV-associated mutations remain detectable in approximately 23% of peripheral blood malignant T cells [62]. These findings support a model in which malignant clones accumulate mutations during a skin-resident phase under environmental exposure, followed by re-entry into the systemic circulation and subsequent dissemination.

Recurrent SCNVs directly influence cell cycle regulation and survival through dosage effects. For instance, aberrations involving *TP53* at 17p13.1 are recurrently associated with aggressive disease features [59]. The p53 protein plays a central role in DNA damage sensing and initiation of apoptosis. The frequency of *TP53* loss or single-nucleotide variants increases during large cell transformation (LCT) [63]. When this pathway is impaired, malignant clones show reduced apoptotic responses to DNA damage induced by chemotherapy or radiotherapy, contributing to treatment resistance. In addition, *TP53*-deficient cells may undergo metabolic rewiring that enhances adaptation to microenvironmental stressors such as hypoxia and nutrient deprivation [64]. Collectively, disruption of multiple tumor suppressor networks, including genomic loss of phosphatase and tensin homolog at 10q23.3 and methylation of the fas cell surface death receptor (FAS) promoter, may cooperate to support clonal survival and disease progression [59,65].

### 4.2. Epigenetic Landscape and Chromatin Organization Remodeling

CTCL is characterized by a methylation profile marked by genome-wide hypomethylation together with focal hypermethylation of tumor suppressor gene promoters. Aberrant hypermethylation of promoter Cytosine-phosphate-guanine islands induces chromatin condensation by recruiting methyl-binding proteins and histone deacetylases, leading to broad impairment of cell cycle surveillance and negative regulatory pathways [66]. A representative target of this process is the Cyclin-dependent kinase inhibitor 2A (*CDKN2A*) gene cluster at 9p21.3, where methylation frequency increases with disease progression [67]. As CTCL progresses from early lesions to tumor-stage and LCT-phase disease, increasing methylation levels are associated with greater aggressiveness and poorer prognosis [68,69]. At the molecular level, *CDKN2A* promoter hypermethylation silences p16^INK4a expression, thereby relieving its physiologic inhibition of cyclin-dependent kinases CDK4/6 [59,70]. This leads to constitutive hyperphosphorylation of the retinoblastoma protein (Rb) and subsequent loss of its inhibitory control over the transcription factor E2F transcription factor (E2F) [67]. E2F then activates downstream transcriptional programs, allowing malignant T cells to bypass normal cell cycle checkpoints and sustain proliferation [71]. In addition, loss of CD26, a clinical hallmark of SS, is largely attributed to promoter hypermethylation. This silencing may attenuate chemokine-mediated restraints on cell migration, thereby enhancing tissue infiltration and hematogenous dissemination [72].

Histone modifications also contribute to metabolic remodeling and immune evasion by altering nucleosome conformation and chromatin accessibility. As the core catalytic subunit of polycomb repressive complex 2, EZH2 is recurrently overexpressed in primary cutaneous anaplastic large cell lymphoma and in MF with LCT [73]. EZH2-mediated histone H3 lysine 27 trimethylation (H3K27me3) promotes epigenetic silencing of the Th1-type chemokine C-X-C motif chemokine ligand 10 [59]. This alteration restricts recruitment and infiltration of effector CD8+ T cells into skin lesions, facilitating immune evasion within the tumor microenvironment (TME) [73]. EZH2 overactivation may also regulate intracellular reactive oxygen species by inhibiting thioredoxin-interacting protein, thereby reducing oxidative stress-induced apoptosis and promoting malignant T-cell proliferation [73].

Epigenetic remodeling also extends to global reorganization of three-dimensional genomic architecture. Special AT-rich sequence-binding protein 1 (SATB1) maintains lineage identity during normal T-cell development by organizing long-range chromatin interactions [74,75]. In CTCL, SATB1 often undergoes epigenetic inactivation through SUV39H1/2-mediated H3K9me3 and H3K27me3 modifications [76]. Loss of SATB1 expression has several downstream consequences. Reduced phosphatase recruitment is associated with persistent STAT5 activation and upregulation of the anti-apoptotic protein *BCL-2*. In parallel, SATB1 loss induces aberrant expression of skin-homing receptors such as CCR10 and promotes a Th2-lineage shift [74,77,78]. This global chromatin reprogramming may help establish a Th2-polarized phenotype by relieving long-range transcriptional repression of the IL4, IL5, and IL13 gene clusters [79,80].

## 5. Molecular Drivers and Emerging Markers

CTCL progression is sustained by the interaction of multiple signaling networks within malignant T cells and the surrounding microenvironment. Central to this network is the JAK/STAT pathway, which links intrinsic abnormalities and cytokine stimulation to transcriptional programs governing tumor cell survival, phenotypic maintenance, and treatment response. Surface molecules such as CD74, which combine pathobiologic relevance with pharmacologic accessibility, are also emerging as clinically relevant markers.

### 5.1. Mechanisms of JAK/STAT Activation

**Figure 3:**
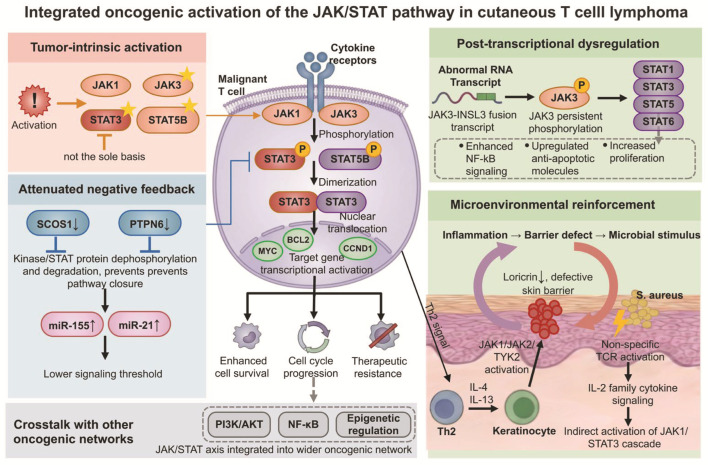
Mechanisms of JAK/STAT signaling activation and tumor progression in malignant T cells. In malignant T cells, JAK/STAT signaling is activated through driver mutations, downregulation of negative regulators, and post-transcriptional dysregulation. In parallel, microenvironmental cues associated with skin barrier dysfunction and *Staphylococcus aureus* further reinforce this cascade. Phosphorylated STAT3 and STAT5B translocate to the nucleus and induce the expression of target genes, including *MYC* and *BCL2*. Together with NF-κB and PI3K/AKT signaling, these processes promote tumor cell survival, proliferation, and therapeutic resistance. AKT: Protein kinase B; BCL2: B-cell lymphoma 2; *CCND1*: Cyclin D1; IL: Interleukin; JAK: Janus kinase; *MYC*: MYC proto-oncogene, bHLH transcription factor; NF-kB: Nuclear factor kappa B; PI3K: Phosphoinositide 3-kinase; *PTPN6*: Protein tyrosine phosphatase non-receptor type 6; RNA: Ribonucleic acid; *S. aureus*: *Staphylococcus aureus*; SOCS1: Suppressor of cytokine signaling 1; STAT: Signal transducer and activator of transcription; TCR: T-cell receptor; Th2: T helper 2; TYK2: Tyrosine kinase 2. Note: Asterisks indicate proteins harboring somatic gain-of-function mutations.

In CTCL, JAK/STAT pathway activation appears to arise through the integration of intrinsic abnormalities and microenvironmental signals (Figure 3, Table 2). Intrinsic activating alterations in molecules such as JAK1, JAK3, STAT3, and STAT5B can directly enhance signal transduction, although these changes alone do not fully account for pathway dysregulation [81,82]. More commonly, pathway activation is sustained by impaired negative feedback mechanisms. Loss or downregulation of inhibitors such as suppressor of cytokine signaling 1 (SOCS1) and protein tyrosine phosphatase non-receptor type 6 (*PTPN6*) reduces dephosphorylation and degradation of JAK kinases and STAT proteins [83,84]. Concurrently, non-coding RNAs, including miR-155 and miR-21, further suppress the expression of these inhibitory molecules [85]. As a result, malignant T cells require less cytokine stimulation to activate this pathway, allowing signaling to persist even in the absence of strong exogenous input. Constitutively active STAT3 and STAT5 regulate target genes involved in survival, cell cycle progression, and treatment resistance, including B-cell lymphoma 2 (*BCL2*), *BCL2L1*, *MYC*, and *CCND1* [86,87].

Beyond classical genetic alterations, post-transcriptional abnormalities also contribute to persistent activation of this axis. Reports of JAK3-insulin-like 3 (INSL3) fusion transcripts in SS suggest that JAK/STAT dysregulation may arise not only at the DNA level but also through aberrant splicing [88]. This fusion transcript has been associated with persistent JAK3 phosphorylation and downstream activation of STAT1, STAT3, STAT5, and STAT6 [89]. It is also linked to enhanced nuclear factor-κB (NF-κB) signaling, upregulation of anti-apoptotic molecules, and increased proliferative capacity. Pathway activity is further reinforced by the skin microenvironment. Th2 cytokines secreted by malignant T cells, particularly IL-4 and IL-13, act on keratinocytes and activate JAK1/JAK2/tyrosine kinase 2 (TYK2)-related signaling. This suppresses expression of genes within the epidermal differentiation complex (EDC), leading to a reduction in barrier-related molecules such as filaggrin and involucrin [80,81]. Impaired barrier function facilitates the entry of exogenous antigens and microbial products into local tissues, further sustaining inflammatory stimuli [90]. In parallel, *Staphylococcus aureus* colonization and its superantigens may indirectly amplify JAK1/STAT3 signaling through nonspecific TCR activation and IL-2 family cytokine signaling [91,92]. The JAK/STAT axis also shows crosstalk with the phosphoinositide 3-kinase (PI3K)/protein kinase B (AKT), NF-κB, and epigenetic regulatory networks [87,93].

### 5.2. CD74 as a Progression-Related Marker in SS and Advanced MF

Among emerging markers in CTCL, CD74 has attracted increasing interest. Traditionally recognized as an major histocompatibility complex class II (MHC II) chaperone involved in antigen processing and trafficking, CD74 also mediates signaling pathways associated with cell survival and migration [94]. In CTCL, the significance of CD74 lies not only in its aberrant expression but also in its surface accessibility and rapid internalization, features that support its use as a supplementary pathologic marker and potential drug-delivery target [95]. Recent studies have detected CD74 expression across multiple CTCL subtypes, with notable prominence in SS and advanced-stage MF [64]. Immunohistochemistry and single-cell transcriptomic analyses both indicate high CD74 expression in tumor-stage MF and SS [64]. Compared with early-stage disease, expression in advanced cases appears more consistent and may be associated with DNA hypomethylation. CD74 is therefore better regarded as a supplementary marker than as an independent diagnostic criterion [96]. In cases of relapse, progression, or complex immunophenotypes, CD74 can be evaluated as part of the surface profile of the malignant clone in conjunction with histology, staging, and other molecular alterations.

Beyond its diagnostic utility, CD74 is also a promising therapeutic target. Preclinical studies have demonstrated that surface CD74 on CTCL cells exhibits rapid receptor internalization, facilitating the intracellular delivery and payload release of the antibody-drug conjugate (ADC) STRO-001 [95]. In several CD74-positive CTCL cell lines, STRO-001 induces cell death and triggers poly(ADP-ribose) polymerase cleavage [64,96]. In xenograft models, it causes tumor regression and produces sustained inhibitory effects in some settings. Its activity does not appear to require very high antigen expression, suggesting that the value of CD74 as an ADC target reflects both antigen accessibility and rapid receptor turnover and internalization. For advanced CTCL, particularly in the setting of *TP53* loss or dysfunction where treatment options are limited, STRO-001 retains antitumor activity in cell lines harboring deleterious *TP53* alterations and shows sustained effects in vivo [64]. These findings suggest that its cytotoxic effects may not depend entirely on an intact p53-mediated apoptotic program, supporting its potential utility in resistant or progressive disease.

## 6. Cellular Interactions Within the Cutaneous Microenvironment

**Figure 4:**
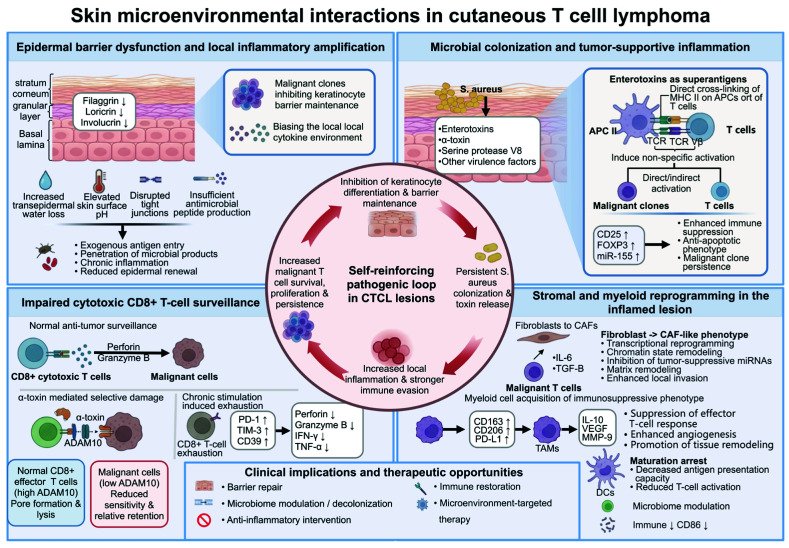
Pathogenic mechanisms and the tumor microenvironment in cutaneous T-cell lymphoma. Epidermal barrier dysfunction in cutaneous T-cell lymphoma is associated with reduced filaggrin, loricrin, and involucrin expression, leading to increased transepidermal water loss, altered surface pH, disrupted tight junctions, and reduced antimicrobial peptide production. These changes facilitate antigen entry, microbial product penetration, and chronic inflammation. In parallel, *Staphylococcus aureus* colonization and toxin release impair keratinocyte differentiation and barrier maintenance, while enterotoxins acting as superantigens promote non-specific T-cell activation and support persistence of malignant clones. Antitumor surveillance is further limited by impaired CD8+ cytotoxic T-cell function and exhaustion. Within the inflamed lesion, fibroblasts and myeloid cells undergo phenotypic reprogramming, contributing to immune suppression and tissue remodeling. Together, these processes form a self-reinforcing pathogenic loop that supports malignant T-cell survival, proliferation, immune evasion, and local disease progression. ADAM10: A disintegrin and metalloproteinase domain-containing protein; APC: Antigen-presenting cell; CAF: Cancer-associated fibroblast; CD: Cluster of differentiation; CTCL: Cutaneous T-cell lymphoma; DCs: Dendritic cells; FOXP3: Forkhead box P3; IFN-γ: Interferon gamma; IL: Interleukin; MHC II: Major histocompatibility complex class II; miR-155: microRNA-155; MMP-9: Matrix metalloproteinase 9; PD-1: Programmed cell death protein 1; PD-L1: Programmed death-ligand 1; *S. aureus*: *Staphylococcus aureus*; TCR: T-cell receptor; TCR Vβ: T-cell receptor variable beta chain; TGF-β: Transforming growth factor beta; Th2: T helper 2; TIM-3: T-cell immunoglobulin and mucin-domain containing-3; TNF-α: Tumor necrosis factor alpha; VEGF: Vascular endothelial growth factor.

The skin microenvironment plays a central role in the pathologic progression of CTCL (Figure 4). As the disease progresses from early lesions to tumor-stage or erythrodermic disease, epidermal barrier disruption, persistent local inflammation, and cutaneous dysbiosis form a mutually reinforcing pathologic circuit. Malignant clones and associated immune cells impair keratinocyte differentiation and barrier maintenance, thereby facilitating colonization by opportunistic pathogens. These organisms release virulence factors that further alter the local immune landscape and promote tumor cell survival and immune evasion.

### 6.1. Epidermal Microenvironment Remodeling and Epidermal Barrier

Epidermal barrier impairment is a key component of microenvironmental dysregulation in CTCL. The integrity of the stratum corneum and stratum granulosum depends on coordinated expression of genes within the EDC, including filaggrin (*FLG*), loricrin (*LOR*), and involucrin, which contribute to cornified envelope formation, keratinization, and skin hydration homeostasis [80]. CTCL lesions frequently show downregulation of *FLG* and *LOR*, accompanied by increased transepidermal water loss, elevated surface pH, and impaired osmotic barrier function [97,98]. Disrupted tight junctions and insufficient production of antimicrobial peptides further weaken the physical and chemical defenses of the skin [97]. These alterations permit entry of exogenous antigens, microbes, and their metabolites into local tissues, thereby promoting chronic inflammation and microenvironmental remodeling.

Changes in the local immune landscape also contribute to this process. As disease progresses, the cytokine profile shifts toward immunosuppression and chronic inflammation, which impedes keratinocyte differentiation and reduces epidermal renewal and repair capacity [90]. Reduced expression of antimicrobial molecules such as β-defensin and cathelicidin LL-37 further compromises control of the skin surface microbiota [97]. The resulting barrier defects therefore reflect not only tissue damage but also a permissive state for subsequent microbial colonization and virulence factor-mediated immune dysregulation.

### 6.2. Microbial Colonization and Maintenance of Tumor-Associated Inflammation

In advanced CTCL, colonization by *Staphylococcus aureus* is markedly increased and may represent not only a secondary infectious event but also a factor contributing to disease maintenance and progression [97,99]. *S. aureus* influences the lesional microenvironment through enterotoxins, α-toxin, serine protease V8, and other virulence factors, thereby affecting malignant T-cell expansion, the functional state of surrounding immune cells, and local epidermal injury [100]. Persistent bacterial burden and exposure to virulence factors amplify pre-existing inflammatory responses and exacerbate barrier damage.

Among these factors, enterotoxins act as superantigens that bypass canonical antigen processing and presentation by directly cross-linking MHC II molecules on antigen-presenting cells with T-cell receptor Vβ regions, inducing broad, non-antigen-specific T-cell activation [91]. In CTCL, this mechanism may act directly on malignant clones with compatible receptor profiles or indirectly create a pro-survival inflammatory environment by activating benign reactive T cells within lesions [101,102]. Following superantigen stimulation, local cells upregulate molecules such as CD25, forkhead box P3, and miR-155, thereby enhancing immunosuppressive and anti-apoptotic phenotypes that support persistence of the malignant clone [103]. Clinical observations suggest that decolonization therapy targeting toxin-producing strains can induce lesional remission and partially restore expression of barrier-related proteins in some patients [102]. These findings raise the possibility that reducing microbial burden may interrupt the pathologic cycle linking barrier damage, microbial colonization, and persistent inflammation.

### 6.3. Impaired Cytotoxic T-Cell Immunosurveillance and Clinical Implications

In CTCL lesions, normal CD8+ cytotoxic T cells serve as a major effector population that limits expansion of malignant clones. They mediate clearance of malignant T cells by recognizing tumor-associated antigens presented via MHC I molecules and releasing perforin and granzyme B [103]. Persistent bacterial virulence factors in the microenvironment can weaken this defense system both quantitatively and functionally. Specifically, α-toxin binds ADAM10 on the cell surface, forming transmembrane pores that induce ionic imbalance and trigger necrosis or apoptosis [104]. Research suggests that normal CD8+ effector and memory T cells are more sensitive to α-toxin, whereas malignant CTCL cells are relatively resistant [105]. This relative resistance may reflect epigenetic downregulation of ADAM10 in malignant cells, which reduces available toxin-binding sites. By contrast, normal CD8+ T cells maintain higher ADAM10 expression and may therefore be more susceptible to preferential clearance in colonized environments. Beyond direct killing, α-toxin also inhibits the cytotoxicity of antigen-specific CD8+ T cells, reducing their capacity to lyse tumor cells [105].

Chronic superantigen exposure and an inhibitory microenvironment also promote functional exhaustion of the remaining CD8+ T cells. CD8+ T cells in advanced MF and SS lesions often exhibit increased expression of PD-1, TIM-3, and CD39, accompanied by decreased production of perforin, granzyme B, interferon-γ, and tumor necrosis factor-α [91]. In the setting of limited local survival signals, these cells become more sensitive to Fas/FasL-mediated pro-apoptotic stimuli. Malignant T cells may further promote clearance of infiltrating reactive CD8+ T cells through upregulation of *FASL* expression [106]. Microbial-driven immune escape in CTCL therefore involves not only enhanced inflammation but also superantigen-mediated chronic functional remodeling and α-toxin-mediated selective killing, which together weaken antitumor immunosurveillance by normal CD8+ T cells.

In addition to direct inhibition of normal CD8+ T cells, stromal and myeloid cells within CTCL lesions undergo functional reprogramming under persistent inflammatory stimulation, further consolidating local immune escape. In the dermis, IL-6 and transforming growth factor-β secreted by malignant T cells can induce fibroblasts to acquire a cancer-associated fibroblast phenotype [107]. This process involves remodeling of transcriptional programs and chromatin states, with suppression of tumor-suppressive microRNAs contributing to matrix restructuring and local infiltration by malignant cells [108]. Myeloid cells also gradually acquire an inhibitory phenotype. As disease progresses, the local cytokine environment induces a shift in tumor-associated macrophages toward an M2-like state, characterized by elevated expression of CD163, CD206, and PD-L1 [109,110]. These cells not only suppress effector T-cell responses through IL-10 secretion but also release vascular endothelial growth factor and matrix metalloproteinase 9, promoting angiogenesis and tissue remodeling [109]. Dendritic cells may likewise exhibit impaired maturation under persistent microenvironmental stimulation, with insufficient expression of the co-stimulatory molecules CD80 and CD86, thereby weakening antigen presentation and T-cell activation [111].

Immune escape in CTCL lesions therefore reflects a local inhibitory network involving bacterial virulence factors, malignant T cells, stromal cells, and myeloid cells, rather than a single cell population. This framework suggests that management of advanced CTCL may benefit from approaches that address not only inflammatory control but also epidermal barrier repair, microbial burden, and restoration of antitumor immunity, thereby expanding therapeutic options for refractory disease.

## 7. Targeted Therapy in Cutaneous T Cell Lymphoma: Translational Strategies and Resistance Mechanisms

As molecular stratification in CTCL continues to evolve, treatment strategies have shifted from non-specific cytotoxic therapies toward targeted interventions based on surface antigens and signaling dependencies. Current research suggests that the durable benefit of targeted therapy depends not only on the presence of the target but also on clonal selection under therapeutic pressure, antigen expression stability, and the activation of compensatory survival pathways.

### 7.1. Acquired Resistance to Anti-CCR4 Therapy: Antigen Loss and ADCC Failure

Mogamulizumab is a humanized defucosylated monoclonal antibody targeting CCR4, used for relapsed or refractory MF and SS [112]. CCR4 is constitutively expressed on the surface of malignant T cells in multiple CTCL subtypes, particularly in tumor clones with a Th2 bias and skin-homing features [113]. The drug eliminates CCR4-positive tumor cells by enhancing antibody-dependent cellular cytotoxicity (ADCC) [114]. Although mogamulizumab improves progression-free survival in some patients, disease progression following treatment indicates that resistance is frequently associated with alterations in the target antigen [115].

Studies suggest that CCR4 antigen loss is a major mechanism of mogamulizumab resistance. Marked downregulation or loss of CCR4 protein expression is observed in patients with post-treatment progression, leading to insufficient drug binding and subsequent attenuation of the ADCC effect [116]. The molecular basis for this includes acquired copy number loss at the CCR4 locus and transcriptional silencing via promoter hypermethylation. Beyond expression changes, mutations in the extracellular N-terminus and transmembrane regions of CCR4 can also impair antibody recognition. Structural analysis reveals that mogamulizumab primarily recognizes epitopes in the juxtamembrane region of the CCR4 extracellular N-terminus, where amino acid residues 14 to 24 form the critical binding interface [114]. Mutations such as L21V reported in resistant patients can alter local conformation and reduce epitope recognition; consequently, the ADCC response remains suboptimal even at high drug concentrations [116]. During treatment, clones with high CCR4 expression are preferentially eliminated, while subclones with low expression or relevant mutations gradually acquire a survival advantage [4]. Collectively, CCR4 copy number loss, critical epitope mutations, and transcriptional silencing form a continuous resistance cascade characterized by “antigen loss—impaired recognition—ADCC failure.”

### 7.2. Translational Application of JAK and HDAC Inhibition: Stratified Therapy and Combination Interventions

The clinical value of JAK inhibitors in CTCL is subtype-dependent and is most clearly defined in SPTCL [117]. SPTCL is frequently associated with germline *HAVCR2* mutations; impaired TIM-3 function leads to dysregulated inflammation and persistent activation of cytokine axes, thereby increasing dependence on JAK/STAT signaling [14]. Systematic reviews show that JAK inhibitors such as ruxolitinib, tofacitinib, and abrocitinib achieve objective response rates (ORR) of 75–100% in SPTCL, higher than the response levels in MF or SS [117]. In advanced MF/SS, the ORR for single-agent JAK inhibitors typically ranges from 11 to 45%, suggesting that these agents are more suitable for specific subtypes with defined driver backgrounds.

In contrast, a major limitation of HDAC inhibitors is the difficulty in maintaining sustained single-agent efficacy. When HDAC inhibitors such as romidepsin are used in CTCL, secondary resistance is often accompanied by upregulation of ErbB family receptors, particularly compensatory survival signaling mediated by epidermal growth factor receptor and ErbB2 [118]. Consequently, the combination of the pan-ErbB inhibitor afatinib and romidepsin has been proposed as a strategy to overcome resistance. Research indicates that afatinib blocks ErbB receptors and their downstream PI3K/AKT and Mitogen-activated protein kinase/Extracellular signal-regulated kinase survival signals [119]. When combined with romidepsin, it enhances the expression of pro-apoptotic molecules and downregulates anti-apoptotic factors such as *BCL-2* and survivin. In xenograft models, this combination delays tumor growth and produces sustained inhibitory effects [118]. Overall, translational research in CTCL is shifting from single-target inhibition toward stratified and combination interventions based on resistance mechanisms.

## 8. Diagnostic Limitations and Future Directions

Although the WHO-HAEM5 classification incorporates molecular parameters into the diagnostic framework for CTCL, current diagnostic and staging approaches still face several technical limitations. In peripheral blood assessment, flow cytometry remains the standard method for determining blood involvement; however, its sensitivity is reduced in low-burden disease, particularly at the B1 stage [120]. While loss of CD7 and CD26, as well as abnormal CD4/CD8 ratios, provide useful diagnostic clues, these phenotypic alterations may also occur in reactive inflammatory conditions, in which background lymphocytes can complicate interpretation [121]. TCR-Vβ analysis provides information on clonality, yet its utility is constrained by limited antibody panel coverage [122]. By contrast, T-cell receptor beta constant 1 (*TRBC1*) constant-region restriction may improve detection of occult clones, especially in borderline cases with intermediate phenotypes; nonetheless, standardization of *TRBC1*-based assessment in CTCL staging still requires prospective validation [121].

Another staging limitation is under-recognition of occult lymph node involvement. Another staging limitation is under-recognition of occult lymph node involvement. Traditional staging relies heavily on clinical palpation, which may miss deep lymphadenopathy even in patients without palpable nodal disease [123]. Studies indicate that routine imaging in early MF leads to upstaging in approximately 14% of cases, suggesting that reliance on palpation alone may underestimate disease extent [124]. PET/CT not only helps distinguish reactive lymphadenopathy from nodal tumor involvement but also provides critical metabolic information for evaluating large cell transformation and response to systemic therapy [125].

In addition, much of the evidence guiding CTCL diagnosis, risk stratification, and management is derived from retrospective studies and is therefore vulnerable to referral bias, sample heterogeneity, and inconsistent testing standards [126]. Future research should prioritize multicenter prospective validation based on standardized flow-cytometric gating, clearly defined imaging indications, and refined molecular stratification. Single-cell sequencing and multi-omics integration are expected to improve resolution of subclonal architecture, minimal residual disease, and microenvironmental interactions [127]. From a therapeutic perspective, microbiome-informed precision approaches may offer a complementary avenue for risk assessment and optimization of combination strategies [102].

## 9. Conclusions

CTCL is a biologically heterogeneous disease shaped by dynamic interplay among cellular origin, clonal evolution, genomic alterations, epigenetic remodeling, microenvironmental interactions, and selective pressure imposed by therapy. Current evidence indicates that MF and SS differ not only in clinical presentation but also in underlying pathobiology, including homing behavior, molecular drivers, and patterns of systemic dissemination. Signaling pathways such as JAK/STAT, emerging markers such as CD74, microbial-driven immune escape, and antigen loss after anti-CCR4 therapy collectively provide important insights into CTCL biology and therapeutic response. Diagnosis and management of CTCL are shifting from empirical assessment toward an integrated model incorporating pathology, molecular stratification, and dynamic monitoring. Integration of prospective cohorts, single-cell sequencing, multi-omics approaches, and microenvironment-directed interventions will be essential for refining early diagnosis, staging, resistance assessment, and precision therapeutic strategies, thereby strengthening the basis for risk stratification and individualized management in CTCL.

## Figures and Tables

**Table 2:** Integrated Mechanisms of JAK/STAT Activation in CTCL.

Category	Representative Events	Key Molecules	Biological Consequence	Clinical/Translational Implication
Tumor-intrinsic activation	Activating alterations directly enhance signal transduction	JAK1, JAK3, STAT3, STAT5B	Increased JAK/STAT signaling output in malignant T cells	Supports the role of the JAK/STAT axis as a central therapeutic target; indicates pathway activation involves more than canonical driver lesions
Attenuated negative feedback	Loss or downregulation of inhibitory regulators weakens dephosphorylation and degradation	SOCS1, *PTPN6*	Failure of pathway shutoff; sustained signaling even under low-intensity cytokine stimulation	Suggests persistent activation may result from defective feedback control rather than strong upstream stimulation
Non-coding RNA reinforcement	Oncogenic non-coding RNAs further suppress negative regulators	miR-155, miR-21	Lowered signaling threshold and prolonged pathway activity	Highlights post-transcriptional regulation as an additional layer of pathway maintenance and a source of biomarkers
Downstream transcriptional output	Constitutively activated STAT proteins induce gene transcription	STAT3, STAT5; *BCL2*, *BCL2L1*, *MYC*, *CCND1*	Enhanced cell survival, proliferation, and therapeutic tolerance	Provides a mechanistic basis for aggressive behavior and resistance phenotypes
Post-transcriptional dysregulation	Abnormal RNA processing activates the pathway independently of DNA alterations	JAK3-INSL3 fusion transcript	Persistent JAK3 phosphorylation and downstream STAT activation; enhanced NF-κB signaling	Broadens the spectrum of actionable molecular abnormalities beyond simple mutations
Microenvironmental reinforcement	Th2 cytokines secreted by tumor cells amplify a feed-forward inflammatory circuit	IL-4, IL-13; JAK1, JAK2, TYK2	Suppression of epidermal differentiation; reduction in barrier proteins; persistent local inflammation	Supports the concept that activation is sustained by reciprocal tumor–microenvironment interactions
Barrier disruption–microbial amplification	Barrier dysfunction facilitates entry of antigens/microbes, sustaining inflammatory signaling	Filaggrin, involucrin	Persistent local immune stimulation and reinforcement of tumor-supportive signaling	Suggests that barrier repair interventions may complement pathway-targeted therapy
Microbial/superantigen enhancement	*S. aureus* colonization indirectly intensifies pathway activation	*S. aureus*, superantigens, IL-2 family cytokines	Nonspecific TCR activation and indirect strengthening of the JAK1/STAT3 cascade	Provides a rationale for considering microbial burden and decolonization strategies in advanced cases
Network crosstalk	JAK/STAT axis functions within a broader oncogenic network	PI3K/AKT, NF-κB, epigenetic networks	Signal amplification, pathway cooperation, and stabilization of the malignant phenotype	Supports combination strategies rather than single-axis inhibition alone

## Data Availability

No new data were created or analyzed in this study. Data sharing is not applicable to this article.

## Abbreviations

The following abbreviations are used in this manuscript:

ADC	Antibody-drug conjugate
AD	Atopic dermatitis
ADAM10	A disintegrin and metalloproteinase domain-containing protein 10
ADCC	Antibody-dependent cellular cytotoxicity
AKT	Protein kinase B
ATM	Ataxia-telangiectasia mutated
BCL2	B-cell lymphoma 2
CAF	Cancer-associated fibroblast
CCL	C-C motif chemokine ligand
CCR	C-C motif chemokine receptor
CDKN2A	Cyclin-dependent kinase inhibitor 2A
CLA	Cutaneous lymphocyte-associated antigen
CTCL	Cutaneous T-cell lymphoma
EDC	Epidermal differentiation complex
FAS	Fas cell surface death receptor
FLG	Filaggrin
HSCs	Hematopoietic stem cells
IL	Interleukin
INSL3	Insulin-like 3
JAK/STAT	Janus kinase-signal transducer and activator of transcription
LCT	Large cell transformation
LOR	Loricrin
MF	Mycosis fungoides
MHC	Major histocompatibility complex class
MYC	MYC proto-oncogene, bHLH transcription factor
NF-κB	Nuclear factor kappa-B
ORR	Objective response rate
PI3K	Phosphoinositide 3-kinase
POT1	Protection of telomeres 1
PTPN6	Protein tyrosine phosphatase non-receptor type 6
S. aureus	Staphylococcus aureus
S1PR1	Sphingosine-1-phosphate receptor 1
SCNVs	Somatic copy-number variations
SNVs	Single-nucleotide variations
SOCS	Suppressor of cytokine signaling
SPTCL	Subcutaneous panniculitis-like T-cell lymphoma
SS	Sézary syndrome
TCM	Central memory T cell
TCR	T-cell receptor
TME	Tumor microenvironment
TP53	Tumor protein p53
TRBC1	T-cell receptor beta constant 1
TRM	Tissue-resident memory T cell
UV	Ultraviolet
